# Safety Assessment of Nanomaterials to Eyes: An Important but Neglected Issue

**DOI:** 10.1002/advs.201802289

**Published:** 2019-06-20

**Authors:** Shuang Zhu, Linji Gong, Yijian Li, Haiwei Xu, Zhanjun Gu, Yuliang Zhao

**Affiliations:** ^1^ CAS Key Laboratory for Biomedical Effects of Nanomaterials and Nanosafety Institute of High Energy Physics Chinese Academy of Sciences Beijing 100049 China; ^2^ College of Materials Science and Optoelectronic Technology University of Chinese Academy of Sciences Beijing 100049 China; ^3^ Southwest Eye Hospital Southwest Hospital Third Military Medical University (Army Medical University) Chongqing 400038 China

**Keywords:** eyes, nanomaterials, nanosafety, ocular damage, toxicity

## Abstract

The production and application of nanomaterials have grown tremendously during last few decades. The widespread exposure of nanoparticles to the public is provoking great concerns regarding their toxicity to the human body. However, in comparison with the extensive studies carried out to examine nanoparticle toxicity to the human body/organs, one especially vulnerable organ, the eye, is always neglected. Although it is a small part of the body, 90% of outside information is obtained via the ocular system. In addition, eyes usually directly interact with the surrounding environment, which may get severer damage from toxic nanoparticles compared to inner organs. Therefore, the study of assessing the potential nanoparticle toxicity to the eyes is of great importance. Here, the recent advance of some representative manufactured nanomaterials on ocular toxicity is summarized. First, a brief introduction of ocular anatomy and disorders related to particulate matter exposure is presented. Following, the factors that may influence toxicity of nanoparticles to the eye are emphasized. Next, the studies of representative manufactured nanoparticles on eye toxicity are summarized and classified. Finally, the limitations that are associated with current nanoparticle‐eye toxicity research are proposed.

## 1 Introduction

Nanoparticles have become omnipresent in workplaces and consumer products at this century.^1^, ^2^ The widespread exposure of nanoparticles to workers who make or use them in manufacturing plants is provoking great concerns regarding their toxicity to human body by scientists and the public.^3^ Over the years, many studies have examined the toxicity profile of nanoparticles on main organs such as respiratory tract, lung, brain, liver, kidney, skin, as well as immune system.^4^, ^5^, ^6^, ^7^, ^8^, ^9^, ^10^, ^11^, ^12^, ^13^, ^14^, ^15^ However, in comparison with the flourishing studies carried out to testify nanoparticle toxicity to those organs mentioned above, one especially vulnerable organ, the eye, is always neglected. As a superficial organ, eyes are usually directly exposed to the surrounding environment. Direct contact of eyes with hazardous substance in the environment can lead to ocular damage. According to the statistics provided by Centers for Disease Control and Prevention, about 300 000 workplace eye injuries are sent to emergency room every year in the USA.^16^ The importance of the eye is needless to say as 90% of outside information is obtained via the ocular system. Severe injuries could lead to significant morbidity and even disability. Therefore, with the increasing amount of nanoparticles released from the widespread nanoproduction and nanoapplication, their potential toxicity to the eyes should be well‐studied. U.S. Food and Drug Administration (FDA) had established the guidance for industry: safety of nanomaterials in cosmetic products (Docket number: FDA‐2011‐D‐0489), which provokes the importance of eye safety in this area. The standard eye irritation test established by The Organisation for Economic Co‐operation and Development (OECD) for the testing of chemicals is used as the standard to measure nanomaterials eye toxicity. The safety use of nanomaterials to eyes surely has received certain attention internationally but the studies about the toxicity effect of nanomaterials on eyes are still at their early stage.

Due to its small size, the toxicity effects of nanomaterials on ocular surface have their own characteristics when compared with mechanical or chemical‐induced injuries.^17^, ^18^, ^19^, ^20^ As there are a series of anatomical barriers on the eyeballs, the particles with large size are excluded out of the ocular surface with blinking and washing away by tear film. However, the small size of nanoparticles ensures the close contact with ocular surfaces, anchoring to the cornea for longer residence time, penetrates the barriers of ocular surface, and reaches posterior segments of the eye.^21^ It demonstrated that the size of the nanoparticles determines the speed and amount to penetrate the ocular surface, which makes the small particles hard to be washed out.^22^ Therefore, nanosized particles are well tolerated and more easily to migrate across epithelial barrier to cause cytotoxicity and inflammatory response.^21^, ^22^, ^23^, ^24^, ^25^ Once entering eyes, nanoparticles may subsequently induce cellular toxicity as well as systematic immune response not only at ocular surface, but also to lens, retina, or even optic nerve and macula. For example, studies have shown that metal‐containing nanoparticles, after entering the eyes, could be conveyed into the nasal cavity through the nasolacrimal duct by drainage from the eye socket, which subsequently enter the central nervous system via the eye–nose–brain route.^25^ These reports clearly showed that the mechanism of nanomaterials toxicity effects on eyes is quite different from other materials and therefore more attention should be received to this important field.

Previous studies have reported many workplace related eye toxicity such as cosmetic/personal care products, pesticide exposure at agricultural sector, hazardous chemicals at industrial place, as well as environmental particles such as building products, air pollutions (PM 2.5), or volcano particles.^17^, ^18^, ^26^, ^27^, ^28^, ^29^, ^30^, ^31^, ^32^, ^33^, ^34^ However, to our best knowledge, a review focus on the summarization of toxicity effects of nanomaterials to eyes is still rare. Herein, instead of covering every single nanomaterial, we choose some representative manufactured nanomaterials from the list made by the Working Party on Manufactured Nanomaterials (WPMN) of the OECD^35^ and classify them into three categories: metal nanomaterials, metal oxide nanomaterials, and carbon nanomaterials. We hope this review could give a general introduction of nanoparticles toxicity to eyes, and at the same time provoke more incisive studies in this important field.

## 2 Nanoparticle Caused Ocular Syndromes

**Scheme 1:**
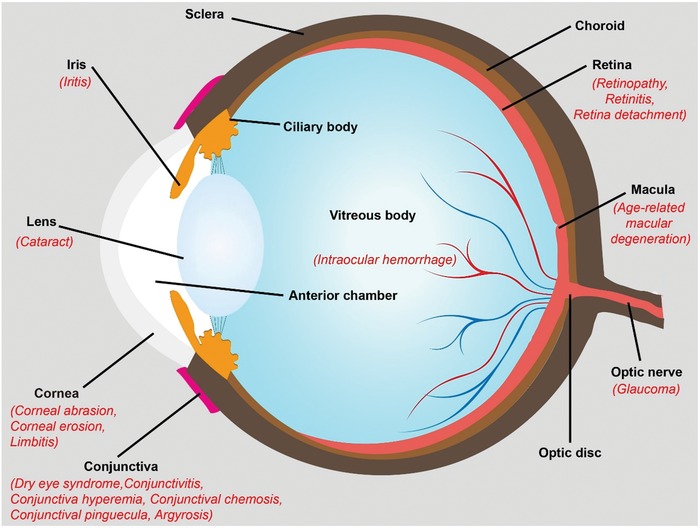
Typical eye disorders caused by nanoparticle exposure.

In this chapter, we give a brief description of ocular anatomy and typical ocular disorders with a focus on occupational or environmental particulate materials exposure. Although the ocular surface is the foremost layer that contacts with nanomaterials, once nanoparticles are absorbed into eyes, toxic effect could also be induced within the eye (such as iris and lens) or inner surface of retina, optic nerve, and macula (**Scheme** 1).^36^, ^37^ What is worth to be noted here is that some symptoms mentioned below may have not yet found in nanomaterial‐related workplace. However, considering the pathogenesis of nanoparticle to eyes, these symptoms might also be induced by nanoparticles and need to acquire equal attention for future study.

### 2.1 Nanoparticles Caused Damage on Eye Surface

Most of the researches regarding the direct influence of nanoparticles on eyes focus on their damage to eye's surfaces. The ocular surface includes two major parts: the cornea and the conjunctiva. Different from other parts of the body covered by skin, the ocular surface is covered with a thin layer of tear film that is supported by lacrimal and accessory lacrimal gland, conjunctival goblet cells, Meibomian gland, and glands of Zeis and Moll.^38^, ^39^ It is proposed that the potential mechanism of ocular symptoms caused by the dysfunction of any ocular surface component is inflammation. The introduction of foreign substances on the epithelial surface could induce a series of inflammatory events, which may subsequently cause tear film instability as well as cell damage.^40^, ^41^, ^42^ Common clinical features of ocular surface inflammation include dryness, burning, itching, gritty eyes, conjunctival injection, dilatation of conjunctival vasculature, conjunctival chemosis, limbitis, redness, and swelling of eyelids.^43^

#### 2.1.1 Dry Eye Syndrome

Also known as keratoconjunctivitis sicca, it is an inflammatory disorder that affects ocular surface and lacrimal gland.^44^ It happens when either the eye produces deficient tear or when tear evaporates too quickly.^45^ The common symptoms usually include feeling of dryness, grittiness, and burning; further complications associated with dry eye syndrome are conjunctivitis and keratitis. Study has shown that particulate matters such as TiO_2_ are more harmful to dry eyes when compared with normal eyes.^46^

#### 2.1.2 Conjunctivitis

Also known as pink eyes, it is an inflammation or swelling of the conjunctiva. People with conjunctivitis may experience pink discoloration, gritty, itching, burning feeling of one or both eyes, excessive tearing, and swollen eyelids. Conjunctival hyperemia and ocular discharge are common symptoms of conjunctivitis. It is reported that the particulate matters could increase the risk of allergic conjunctivitis.^47^

#### 2.1.3 Conjunctival Hyperemia

Also known as conjunctival injection, it is one cause of eye redness, which results from dilation of conjunctival vessels and increased blood flow. Particulate matter triggered allergic cascade (by releasing histamine) could result in vasodilation and increased blood flow.^48^

#### 2.1.4 Conjunctival Chemosis

Chemosis is the swelling of the conjunctiva, resulting from the extravasation of plasma.^48^

#### 2.1.5 Conjunctival Pinguecula

It is a common type of conjunctival degeneration, which is usually associated with yellow‐white deposit on conjunctiva near to limbus. It is found that people with silicosis have a high risk for conjunctival pinguecula in silica exposure environment.^49^

#### 2.1.6 Argyrosis

Also know as argyria, it is caused by the gradual accumulation of silver products in eyes. Argyrosis particularly indicates the argyria of the conjunctiva. Once developed argyrosis, conjunctiva may turn blue‐gray.

#### 2.1.7 Corneal Abrasion

Scratched cornea is a common ocular injury that results from damage or loss of corneal epithelial cells. The usual symptoms of corneal abrasion are pain/gritty feeling, red eyes, and hypersensitivity to light. Several studies have shown that nanoparticle exposure could cause destruction to corneal epithelium cells through different mechanisms such as cell membrane damage, mitochondrial dysfunction, or cell death.^50^

#### 2.1.8 Corneal Erosion

The common symptom is similar to the initial state of corneal abrasion with tearing on awakening. Its recurrence may occur years after a corneal abrasion.

#### 2.1.9 Limbitis

The inflammation of limbus. Limbus, the special site where corneal epithelial stem cells reside, is the border of the conjunctiva and cornea.

#### 2.1.10 Uveitis

Also called iritis, it is the inflammation of uvea, which includes iris, ciliary body, and choroid. The common symptoms are redness or burning of eyes, blurred vision, sensitivity to light, and so on. Bacteria, virus, or particulate matter‐induced over‐reactive immune system could be the causes of this disease.^51^

### 2.2 Nanoparticles Induced Damage on Lens

It has been well stated that cigarette smoking, solid fuel (such as wood) combustion, and metal iron could cause damage to lens. Smoking is found to be related with metal accumulation such as cadmium which can cause oxidative DNA damage or per oxidative damage to lens cell membrane for triggering cataractogenesis;^52^ the alteration in metal ions may be a contributor to cataract formation by inducing oxidative stress, inhibiting antioxidant pathways, or modifying the structure/formation of lens extracellular matrix;^53^ an iron intraocular foreign body sedimentation may lead to siderosis which may cause cataract and lens discoloration.^54^ It is proposed that metal irons could cause oxidative damage to lens through the metal catalyzed Fenton reaction.^55^

#### 2.2.1 Cataract

Cataract is a clouding of the internal lens mainly caused by aging, radiation, genetics, complication of other disease, etc.^56^ People who have cataract often suffer from diminished ability in vision. The common symptoms include reduced color intensity, blurry vision, and tough seeing with bright light and at night.^57^

### 2.3 Nanoparticles Induced Damage on Retina

As mentioned above, particulate materials could induce inflammatory factors production, which further elicit systematic inflammatory response with enhanced cytokine production.^58^, ^59^, ^60^ Therefore, we could also speculate that the induction of chronic exacerbation of immune response could cause damage to layers of retinal vessels, degeneration of retinal cells, and neovascularization.^61^ For instance, studies showed that gold, silver nanoparticles, or multiwalled carbon nanotubes (MWCNT) could cause increased cell apoptosis and oxidative stress in animal retinal cells or tissue.^62^, ^63^ In addition, it is reported that the particulate materials could increase serum homocysteine level, and further reduce the ocular blood flow velocity.^64^ Besides, excess iron ions dissolved from iron nanoparticles in eyes are associated with retinal detachment, age‐related macular degeneration, and intraocular hemorrhage;^54^, ^65^, ^66^ ZnO nanoparticles could increase retinopathy.^67^

#### 2.3.1 Retinopathy

Retinopathy is the damage to the retina, which may lead to vision impairment or even vision loss. It usually refers to retinal vascular damage or retina damage induced by abnormal blood flow.^68^ Retinopathy is usually the complication from diabetes, which is also called diabetic retinopathy.

#### 2.3.2 Retinitis

It is the inflammation of the retina which can cause permanent damage to retina. People with retinitis may experience slow night/peripheral vision loss or eventually blindness. Iron is reported to cause retinitis.^69^

#### 2.3.3 Retina Detachment

A disorder in which the neural retina separates from the underneath retinal pigment epithelium.^70^ People with retinal detachment may experience symptoms such as flashes of light, visual floaters, shadow at peripheral vision, or central visual loss.^70^

#### 2.3.4 Age‐Related Macular Degeneration

It is one specific kind of retinopathy which affects the macula, the structure that is responsible for the high‐acuity vision. Although it may not cause total blindness, it gets worse as ageing.

#### 2.3.5 Intraocular Hemorrhage

It is a disorder of bleeding into the eyeball. It may be caused by the abnormal vessels or damage of normal vessels. The common symptoms include floaters and visual loss. It is also a potential complication of proliferative diabetic retinopathy or retina vasculitis.^71^, ^72^

### 2.4 Nanoparticle Induced Damage on Optic Nerve

Upon nanoparticles exposure, ocular surface including corneal and conjunctival epithelium provides protection against their absorption into vitreous humour.^36^ Vitreous humour is located at the anterior and posterior chambers of the eye, which is in close contact with retina, optic nerve, and macula.^37^ Therefore, particulate materials from vitreous humour also injure optic nerves. In addition, it has shown that fine particles also result in increasing intraocular pressure under constant exposure,^73^ and cause damage to optic nerves.

#### 2.4.1 Glaucoma

A disease that causes damage to the retinal ganglion cells and optic nerve, usually caused by the high intraocular pressure. Particulate matters might cause glaucoma by “clogging” the trabecular meshwork and interfere the outflow channels.^74^ It could cause diminished peripheral vision or even blindness.

## 3 Nanoparticle Toxicity Influencing Factors on Eyes

Current studies regarding nanoparticle eye toxicity factors mostly put their focus only on size or exposure time, however, there are much more distinguished parameters that may influence the eye toxicity of nanoparticle, due to their unique physiochemical properties. Besides, the hazard of one specific nanoparticle is often influenced by several kinds of properties. The resulted toxicity is usually due to a combination of the properties instead of one single factor. The important factors that influence toxicity of nanoparticles toward eye are summarized as follows. These factors could also be used as a reference for future nanoparticle eye toxicity studies.

### 3.1 Chemical Components and Nanoparticle Chemistry

The two notions of nanoparticle chemistry and chemical components are different. Chemical components refer to the chemical elements that nanoparticle contain; although two kinds of nanoparticle have the same components, they may possess different chemical or crystalline structure. These two profiles are both critical in determining nanoparticle toxicity. Nanomaterials usually contain more than one chemical. Some are of great toxicity due to their chemical components such as heavy metal or other kind of poisonous elements.^75^ For instance, the high toxicity of ZnO nanoparticles is probably due to the release of toxic Zn ion in the lysosomes after internalized into cells.^76^ Different metals of metal oxide nanoparticles can cause different levels of toxicity due to their ability to cause reactive oxygen species (ROS) generation.^77^

Besides, the chemistry of nanoparticle may influence toxicity profile in a few ways, such as affecting cellular uptake, subcellular localization, and oxidative stress.^78^ For example, there are two forms of TiO_2_, rutile and anatase. Although they contain same chemical composition, due to the different crystalline structure, rutile nanoparticles exhibited high oxidative damage to human bronchial epithelial cells in the absence of photoactivation, while anatase nanoparticle at the same size did not.^79^

### 3.2 Size and Shape

Size can influence toxicity in many ways. It is known that as nanoparticle size decreases, the surface area increases exponentially.^80^ Thus more chemical molecules may attach to the big surface, which further increase its surface reactivity as well as toxic effects.^81^ For example, SiO_2_ nanoparticles with diameters less than 40 nm show a higher toxicity to human corneal epithelial cells than those bigger than 50 nm.^50^ In addition, smaller sized nanoparticles are more easily to migrate across epithelial barrier to cause cytotoxicity and inflammatory response.^82^, ^83^

Apart from size, shape is another factor to influence nanoparticle toxicity. For instance, nanoparticles with higher aspect radio tend to be more toxic.^84^ It is indicated that fiber‐shaped nanoparticles are more toxic than spherical‐shaped nanoparticles of the same chemical composition.^85^ Studies also show that compared to amorphous carbon black (spherical particles), carbon nanotubes with long aspect ratio are more toxic.^86^, ^87^, ^88^ In addition, 2D nanomaterials such as graphene with sharp sheets can also exert toxicity by causing damage to cell membrane.^89^

### 3.3 Surface Area/Charge and Modification

The surface of nanoparticle also plays major role in regulating nanoparticle toxicity. The surface area, surface charge, as well as surface modification are all important factors. As mentioned above, as nanoparticles are smaller in size, their surface area becomes bigger correspondingly. When nanoparticles are of same mass, same chemical composition, and same crystalline structure, it is well established that the inflammatory response is proportional to their total surface area.^90^, ^91^, ^92^ Several studies have demonstrated that larger surface area can induce increased reactivity as well as oxidative and DNA damage.^91^, ^93^, ^94^ Additionally, changes in surface charge also result in differences in toxicity of nanoparticles, as it can influence biodistribution as well as cellular uptake efficiency of the nanoparticles.^95^, ^96^, ^97^, ^98^ It is reviewed previously that positively charged nanoparticles can be more easily taken up by cells than negatively or neutrally charged ones, which leads to more inflammatory effects and cell death.^85^ Recently, the oxidation state is also found to be important in some nanomaterials such as GO, where higher oxidation state induces more harmful effects to the eyes.^99^

As it is the surface of nanoparticle that directly interacts with cells and biological materials, surface modification/functionalization can significantly change the bio‐physicochemical properties of nanoparticles such as chemical reactivity, magnetic or optical properties, stability, and biocompatibility.^100^, ^101^, ^102^ These properties are closely associated with the safe or toxic profile of nanoparticles. For example, GO nanoparticles are found to cause damage to ocular surface both in vivo and in vitro, while this toxicity could be alleviated by using proper functionalization with polyethylene glycol (PEG) or antioxidant glutathione (GSH).^99^, ^103^

### 3.4 Dosage and Concentration

Some studies show that the toxicity of nanoparticles is not closely related to nanoparticle mass dose when compared to size differences.^100^, ^104^ For example, it is found that low dose (10 mg m^−3^) exposure to 20 nm TiO_2_ nanoparticles induced much higher long tumor incidence than high dose (250 mg m^−3^) exposure of 300 nm particles.^105^ Also higher concentrated GO exposure is reported to cause damage to human corneal/conjunctiva epithelium cells.^103^ However, one thing should be noted that high concentration of nanoparticles could induce the formation of particle aggregation, which may reduce its toxic effects compared to lower concentrations of the same nanoparticles.^79^, ^106^, ^107^ Besides, several studies also showed that high dose of nanoparticles could expose adverse effects to health.^108^, ^109^, ^110^ Therefore, it is believed that concentration of nanoparticle is another determinant of its toxicity profile.

### 3.5 Behavior in Solvent or Biological Media

Nanoparticles tend to have different behaviors regarding dispersion or agglomeration state in different solvents, which in turn influence their size as well as toxicity profile.^111^ It is indicated that TiO_2_ and ZnO nanoparticles show considerably bigger size in phosphate buffer saline (PBS) than in water.^95^, ^112^ Accordingly, their corresponding toxicity varies in different solvents. Besides, it is also well accepted that nanoparticles display different sizes or agglomeration state in biological media because of the formation of protein corona.^113^ Such protein corona also changes the size and surface properties of nanoparticles, so as to influence their absorption, transportation, as well as toxicity. For instance, the introduction of foetal bovine serum (FBS), which might provide sufficient protein for the formation of protein corona around nanoparticles, was found to alleviate the toxicity of ultrafine SiO_2_ nanoparticles to primary human corneal epithelial cells.^50^

## 4 Ocular Toxicity toward Workplace Nanomaterials

Numerous toxicity studies have demonstrated short‐term associations between different levels of nanoparticle exposure and increased acute morbidity. It is proposed that nanoparticles could cause cellular toxicity mainly by four mechanisms. a) Generation of oxidative stress. For instance, metal nanoparticles such as iron or Au/Ag nanoparticle could cause ROS production and further impact cellular functions (i.e., apoptosis) to cause damage to cells.^62^, ^114^ b) Disruption of cell membrane. Some nanoparticles such as graphene oxide (GO) are reported to cause damage to the membrane of cells due to their sharp sheets.^89^ c) Induction of inflammation response. Once absorbed by cells or entering blood circulation, nanoparticles tend to activate inflammatory responses. Several nanoparticles such as gold, TiO_2_, Fe, GO nanoparticles possess inflammatory nature, which could increase inflammatory cells and proinflammatory cytokine production.^115^ d) Genotoxicity. Due to the typical physicochemical characteristic and large surface area to volume ratio, nanoparticles may induce unpredictable genotoxicity.^116^ It is reported that the long‐term oxidative stress and inflammation caused by nanoparticles could eventually cause DNA damage.^117^, ^118^ Metal nanoparticles such as gold or silver with oxidative nature are found to cause genetic damage.^119^, ^120^ Understanding the mechanism of nanoparticle toxicity, we can better design a proper way to lessen or even avoid their toxic exposure.

Here we present a few commonly manufactured nanomaterials listed from WPMN of the OECD. These important nanomaterials are now widely used in many industrial and commercial products, which may cause the most direct toxicity to the public. Efforts have been put to study their ocular toxicity. Here, we generally classify them into three parts: metal, metal oxide, and carbon nanomaterials.

### 4.1 Metal Nanomaterials

**Figure 1:**
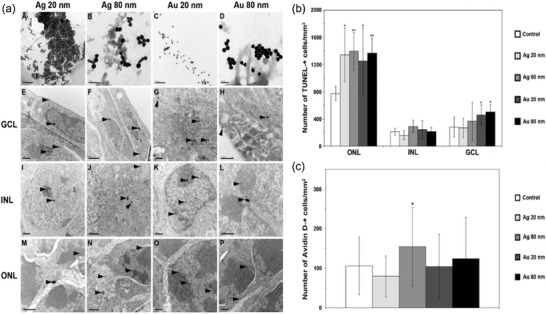
a) TEM‐images demonstrating uptake of Ag and Au NPs in the mouse retina. The top row shows the respective NP structure, i.e., AgNP 20 nm (A), AgNP 80 nm (B), AuNP 20 nm (C), and AuNP 80 nm (D). (E)–(P) All four types of NPs were taken up by the cultured retina and found in all three retinal nuclear neuronal layers, i.e., the GCL, INL, and ONL. Both 20 and 80 nm sized NPs were found either as single NPs (e.g., E, M (upper arrowheads) or as clusters of NPs (e.g., E, I, M (lower arrowheads). The 20 nm sized Ag and Au NPs, respectively, were found in the nucleus (E, G, I, K, M, O), the nucleolus (I, K, O), the mitochondria (G), the cytosol (E, G, M) and in the extracellular space (M). In the majority of cells the largest fraction of the 20 nm sized NPs were located in the nucleus (I, K, O) compared to the fraction of NPs found in the other cell compartments. Notably, all NPs were found both in the eu‐chromatin (e.g., I, K, N) and heterochromatin (e.g., I, K, L, P, M). Ag and Au NPs, sized 80 nm, were detected in the nucleus, but to a lesser extent compared to the 20 nm NPs (F, J, L, N, P) and in the extracellular space (H, L, P). NPs, sized 80 nm, were not detected in the nucleolus and within mitochondria. GCL = ganglion cell layer, INL = the inner nuclear layer, ONL = the outer nuclear layer. Arrowheads show NPs that have been taken up by the retinal cells. Scale bars equal 0.2 mm for images (A–D); 1 mm for (E,F), (I,J), and (M,N); and 0.5 mm for (G,H), (K,L), and (O,P). b) Increased number of apoptotic cells in the mouse retina after exposure to 20 and 80 nm Ag and Au NPs. Graph shows numbers of TUNEL‐positive cells and results are presented as mean ±SD (*n* = 5–8 explants per group). *p < 0.05 compared to control, **p < 0.01 compared to control. Scale bars equal 100 mm. c) Oxidative stress. Graph shows numbers of AvidinD‐positive cells and results are presented as mean ±SD (*n* = 4 explants per group). *p < 0.05 compared to control. Scale bars equal 100 mm. Reproduced with permission.^62^ Copyright 2014, Public Library of Science.

Metallic nanomaterials are among the most widely used nanomaterials in workplace.^4^, ^121^ For instance, silver nanoparticles due to their antimicrobial ability are widely applied in household detergents, antibacterial sprays, apparel, socks, and shoes;^122^, ^123^ ionic Ag and Au can be reduced to their metallic nanoparticles by dissolved organic matter under natural sunlight;^124^ many metal nanoparticles can be spontaneously generated from metal objects, including wire and earrings.^125^ Although bulk metals have been considered to be safe, several research groups have found that their nanoscale particles may exert toxic effects on eyes. For instance, Soderstjerna et al. studied both silver and gold nanoparticles internalization, apoptosis, and oxidative stress using an in vitro cell/tissue culture model of the mouse retina.^62^ Both 20 and 80 nm Ag and Au nanoparticles were used in this study. After 72 h nanoparticle exposure, the researchers found that even low concentration (<0.0065 µg mL^−1^ for 20 nm nanoparticles and <0.4 µg mL^−1^ for 80 nm nanoparticles) of nanoparticles demonstrated undesired effects on the retina cells, such as significant oxidative stress and cell apoptosis (**Figure** 1). The results also revealed that these nanoparticles exerted neurotoxic effects especially on the photoreceptors, which may lead to visual impairment or even blindness. Biswas's group found that the ocular toxicity of Au nanoparticles is closely related to their size, shape, concentration, as well as surface area.^126^ Gold nanoparticles also disrupted Zebrafish ocular development and pigmentation.^127^ In addition, the work of Sriram et al. showed that silver nanoparticle induced cytotoxicity and apoptosis by producing increased ROS in bovine retinal endothelial cells in a size‐dependent manner.^128^ Besides, Kim et al. evaluated the acute eye irritation potential of Ag nanoparticle using New Zealand white rabbits according to OECD test guidelines.^129^ Conjunctival redness, edema, and discharge were observed 1 h after removing the Ag nanoparticles, while no signs of irritation to the cornea, iris, or conjunctiva were found 24, 48, and 72 h after removing the substance. However, Maneewattanapinyo et al. in a study using guinea pigs exposed to 5000 ppm Ag nanoparticles, a grade 1 conjunctivae irritation during first 24 h was observed.^130^ Despite these results, it is also known that long‐term exposure to silver or colloidal silver could cause argyrosis (pigmentation of the eye).^131^, ^132^, ^133^, ^134^ Therefore, the chronic occupational exposure of Ag nanoparticles to eye toxicity may need to be studied at this point.

In contrast to the toxicity study, the eye safety researches of Au and Ag nanoparticles were also studied to some point. Kim et al. once examined whether intravenously administered Au nanoparticle could pass through the blood–retinal barrier (BRB) and cause toxicity in C57BL/6 mice.^24^ They found that 100 nm nanoparticles were not detected in the retina, whereas 20 nm nanoparticles could pass through BRB and affected little cellular viability of retinal endothelial cells and astrocytes. Another safety study also indicated that intravitreal nanogold at concentrations of 67 × 10^−3^ m/0.1 mL and 670 × 10^−3^ m/0.1 mL exhibited no retinal or optic nerve toxicity within 1 month histologic examination.^135^ Additionally, in a series of study made by Gurunathan group, Ag nanoparticles were found to not only inhibit cell survival via PI3K/Akt dependent pathway in bovine retinal endothelial cells,^136^, ^137^ but also inhibit vascular endothelial growth factor and interleukin‐1 (IL‐1) induced vascular permeability via Src dependent pathway in porcine retinal endothelial cells.^138^ These results showed that Ag nanoparticles could be used as therapeutic agents to inhibit the ocular diseases such as diabetic retinopathy.

In addition to heavy metals, other metals such as iron also commonly exist in workplace and can exert potential toxicity to eyes. Previously, it was well reviewed by Dunaief's group that iron could induce ocular siderosis and be associated with a broad range of ocular disease including iris heterochromia, glaucoma, cataract, lens discoloration, retinal detachment, age‐related macular degeneration, as well as intraocular hemorrhage.^54^, ^65^ Recently, Park et al. further examined the toxicity mechanisms of nanosized iron particles in human corneal epithelial cells.^139^ After 24 h incubation, they found that Fe nanoparticles could elevate levels of inflammatory mediator such as nitric oxide, cytokines, and chemokine, increase level of multiple cell death‐related pathway indicators, as well as generate mistranscripted RNA. However, their study was only at in vitro experiment level, and thus further in vivo research is required to investigate the ocular toxicity of iron nanoparticle in a more practical way.

### 4.2 Metal Oxide Nanomaterials

The manufacturing of metal oxide nanoparticles is reportedly done in large production quantities. According to the National Nanotechnology Initiative of America, silica and ceria in the form of ultrafine abrasive particles including nanoparticles are produced each year in thousands of tons scales for precisely polishing silicon wafers.^4^ Until 2012, nearly 1.5 million tons of silica nanoparticles had been placed in global market for agriculture, food, and consumer products such as cosmetics.^140^, ^141^ In recent years, cerium oxide nanoparticles and cerium oxide nanoparticle‐containing materials have intensely been used in polishing glass and jewelry, and in catalytic converters for many industrial and commercial applications.^142^, ^143^ In addition, titanium dioxide and zinc oxide nanoparticles are applied in sunscreens and other cosmetics in a large scale for decades.^144^ Such large‐scale production and use of metal oxide nanoparticles have increased the risk of human exposure to them, while their toxicity study on eyes is only at early stage.

Photoreceptor cells are characterized with super high rate of oxygen metabolism.^145^ Thus, photoreceptor cells are constantly exposed to the adverse effects of oxidative stress and light photons. In blindness causing diseases, including inherited retinal degeneration, diabetic retinopathy, macular degeneration, and retinal detachment, regardless of the initiating pathogeny, the intracellular reactive oxygen species are thought to be generated in either chronic or acute ways and are able to induce cell death.^146^, ^147^, ^148^, ^149^ Due to their redox capacity, cerium oxides are widely used as antioxidant which can reduce oxidative stress in eye.^150^, ^151^, ^152^, ^153^ Notably, McGinnis's group has utilized the antioxidative feature of nanoceria particles to treat different retinal diseases. For example, they found that cerium oxide nanoparticles could prevent ROS‐induced cell death in primary cell culture of rat retina and prevent vision loss caused by light‐induced degeneration of photoreceptor cells.^149^ They also reported that in a model of tubby mutant mice, which generally exhibit inherited retinal and cochlear degeneration, nanoceria could delay photoreceptor degeneration and protect retinal function by upregulating genes related to neuroprotection, survival signal pathways, oxidative stress, antioxidant defense, and key photoreceptor‐specific gene, as well as down‐regulating apoptosis signaling pathways and reducing mislocalization of photoreceptor‐specific proteins.^154^, ^155^ In addition, they also studied the antioxidant as well as neovascularization inhibiting function of nanoceria particles in wet age‐related macular degeneration mouse models (very low density lipoprotein receptor knockout (vldlr^−/−^) mouse).^156^, ^157^ Recently, they further explored the catalytic activity of cerium oxide nanoparticles in another photoreceptor degeneration P23H‐1 rat model.^158^ Upon antioxidant research, several studies also showed that nanoceria particles exert no genotoxic effect on human lens epithelial cells^159^ as well as no toxicity in rat retina using intravitreal injection for a long time.^160^, ^161^

**Figure 2:**
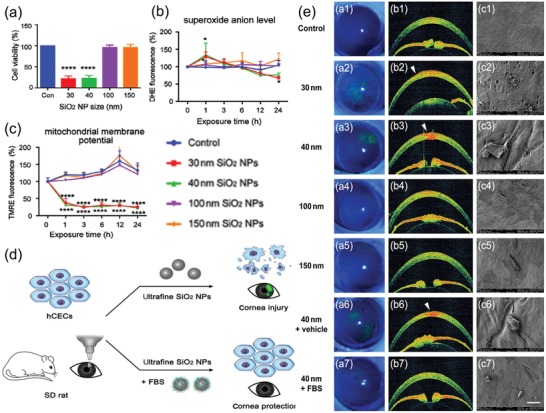
a) Effect of SiO_2_ NPs on cell viability for range of diameters and concentrations. Mean cell viability of hCECs, measured by CCK8 with a microplate reader following treatment with SiO_2_ NPs of diameters 30, 40, 100, and 150 nm, at 100 µg mL^−1^, for 24 h. *n* = 3 per bar; error bars, standard deviation (SD); ****P < 0.0001 by one‐way ANOVA and Tukey's post hoc test. b,c) Damage to hCECs exposed to SiO_2_ NPs with varying size. b) Mean intracellular O_2_^●−^, as measured by dihydroethidium (DHE) fluorescence of hCECs exposed to 100 µg mL^−1^ SiO_2_ NPs of different diameters between 0 and 24 h. c) Mean mitochondrial membrane potential, as measured by TMRE fluorescence of hCECs exposed to 100 µg mL^−1^ SiO_2_ NPs of different diameters for 0–24 h. *n* = 3 per bar; error bars show SD; *P < 0.05, ****P < 0.0001, compared with control. d) Scheme of toxicity of silicon dioxide nanoparticles on the cornea and protein corona as a strategy for therapy. e) Structural corneal damage caused by exposure to SiO_2_ NPs with varying size and therapeutic effect of FBS. a1–a5) Representative images of corneal fluorescein staining under cobalt blue light following exposure to distilled water, and 30, 40, 100 and 150 nm SiO_2_ NPs, respectively. Corneal damage is revealed by green fluorescence. b1–b5) Representative AS‐OCT images following exposure to the same agents in (a1–a5). White arrowheads indicate corneal defects. c1–c5) Representative SEM images following exposure to the same agents as in (a1–a5). Scale bar, 400 mm. Representative images of corneal fluorescein staining (a6, a7), AS‐OCT (b6, b7), and SEM (c6, c7) for cornea treated with vehicle or FBS after 40 nm SiO_2_ NPs, respectively. Reproduced with permission.^50^ Copyright 2018, Elsevier.

In comparison with the huge production and exposure of silica nanoparticles in our society, the study of their eye toxicity is greatly limited. It is well known that the inhalation and retention of crystalline silica may cause severe silicosis, a fibrotic disease of the lungs. However, it was not until 2008 that researchers started studying the effects of silica exposure to the eyes. Yoruk et al. for the first time suggested that eyes can be also considerably affected in the patients with silicosis.^49^ They found that after 40 ± 26 months silica exposure, patients with silicosis exhibited significant conjunctival hyperaemia and pingueculae. Later on, researchers started putting their attention to the ocular toxicity of silica nanoparticles. For instance, Park's group reported several studies that silica nanoparticles of 50, 100, and 150 nm did not induce significant cytotoxicity in cultured human corneal epithelial cell^162^ and human corneal keratocytes.^163^ The in vivo studies using Sprague–Dawley rats also confirmed the safe application of silica nanoparticles to ocular topical administration.^164^ However, a recent study by our group found that ultrafine (30 and 40 nm) SiO_2_ nanoparticles caused toxicity such as cell membrane damage, cell death, and mitochondrial dysfunction to primary human corneal epithelial cells as well as observable corneal injury in Sprague–Dawley rats (**Figure** 2a–d).^50^ With regard to this, it is suggested that the size difference (≤40 nm in our study and ≥50 nm in Park's study) might exhibit different toxicity both in vitro and in vivo. Further study may be needed to find out the comprehensive influencing factors of silica nanoparticle toxicity on eyes, in order to determine the standard safe use of silica nanoparticle. Interestingly, we also found that the ultrafine SiO_2_ nanoparticles induced toxicity could be significantly reduced by the introduction of FBS (Figure 2e,f), likely due to the formation of a protective protein corona formation around the nanoparticles. Although other antioxidants such as GSH, resveratrol, and curcumin were also examined for their ability to prevent SiO_2_ nanoparticle cytotoxicity, none of them exhibited sufficient protective effects. This study provides an alternative clinical treatment (FBS or its derivatives) for reducing corneal toxicity caused by ultrafine particulates.

**Figure 3:**
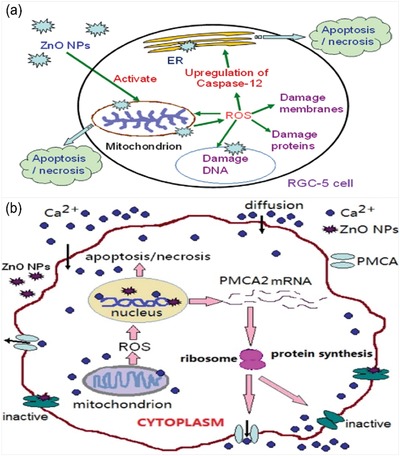
a) Principle of ZnO nanoparticle‐induced the apoptosis/necrosis of RGC‐5 cells. Reproduced with permission.^170^ Copyright 2013, Elsevier. b) Schematic illustration of putative calcium signaling pathway in RGC5 cell death induced by ZnO nanoparticles. The scheme illustrates the possible pathway of intracellular calcium ion elevation mediated by plasma membrane calcium ATPases. Because of exposure to ZnO nanoparticles, the Ca^2+^‐ATPase activity is inhibited, leading to influx of extracellular calcium and elevation of intracellular calcium ion level, then mitochondria produce excessive ROS. The excessive ROS can also decrease the production of PMCA2 at gene and protein levels, inhibit the Ca^2+^‐ATPase activity, and further aggravate the disrupted intracellular calcium homeostasis, finally initiate the cellular apoptosis/necrosis. ER = endoplasmic reticulum. Reproduced with permission.^171^ Copyright 2013, Elsevier.

Another two typical metal oxide nanoparticles, titanium dioxide and zinc oxide, are also common in industrial and commercial products. Generally, primary TiO_2_ and ZnO nanoparticles are 10–20 nm in size, but they are typically used in cosmetics as 30 to 150 nm aggregates.^165^ As size plays an important role in nanoparticle toxicity, different groups measured ocular toxicity using different sizes of TiO_2_ nanoparticles. Previous study showed that whole‐body exposure of airborne TiO_2_ microparticles to rats could induce ocular surface immune system, where the Type 2 helper T‐cell pathway plays a key role.^42^ It resulted in the increasing of interferon‐c (IFN‐c), IL‐4, and IL‐17 levels in anterior segment of eyeball. Several studies showed that TiO_2_ nanoparticles of 20–50 nm did not induce noticeable toxicity to retinal constituent cells as well as retina of C57BL/5 mice and zebrafish.^166^, ^167^ Besides, in an acute toxicity study in rabbits, Warheit et al. found that 129.4 nm fine TiO_2_ nanoparticles could cause very low toxicity and reversible ocular conjunctival redness in rabbits.^168^ However, with repeated exposure, Kim's group found that TiO_2_ of less than 75 nm could induce ocular surface damage, where the area of conjunctival goblet cells decreased after TiO_2_ exposure.^169^ In addition, the ocular surface of dry eyes is also reported to be more vulnerable to TiO_2_ nanoparticles exposure than that of normal eyes (using normal and experimental dry eye rat models).^46^ Apart from TiO_2_ nanoparticles, the ocular toxicity of ZnO nanoparticles is also tested in many researches. For instance, Bi's group utilized rat retinal ganglion cells to study the toxicity effect as well as mechanisms of ZnO nanoparticles. As reported, ZnO nanoparticle could cause cell death via overproducing ROS and caspase‐12, decreasing the expression and activity of plasma membrane calcium ATPase and bcl‐2/caspase‐9, as well as disrupting the intracellular calcium homeostasis (**Figure** 3a,b).^170^, ^171^, ^172^ Moreover, Kim et al. also reported that treatment with 20 nm, negatively charged ZnO nanoparticles could increase retinopathy, which is associated with their local distribution in ocular lesions.^67^

### 4.3 Carbon Nanomaterials

The carbon nanoparticles considered here include fullerenes, carbon nanotubes (both single‐walled carbon nanotubes (SWCNTs) and MWCNTs), and graphene and its derivatives (such as GO or reduced graphene oxide (rGO)). Fullerenes represent a group of nanoparticles comprising the entire of carbon atoms (C*_x_*), which have been widespreadly used in many skin care, cosmetics, and biomedical applications such as bioimaging and tumor treatment.^173^, ^174^ Pristine fullerene C60 is the most common and stable type among fullerene family (such as C70, C76, C90, C28, C36, etc.).^174^ On the other hand, carbon nanotubes are the cylindrical shape of fullerenes. With their unique 1D hollow nanostructure and special characteristics, they are also broadly used in energy storage, molecular electronics, artistic materials, medical and health devices, and many others.^175^ It was reported that the annual global production of carbon nanotubes was running over 100 tons in 2004 and the global revenues from carbon nanotubes were about $230 million with a growth rate of ≈170% in 2006.^176^, ^177^ In addition, graphene and its derivatives are the 2D form of carbon in nature. Since its discovery in 2004, graphene and its derivatives are considered to be the new revolutionary materials and are widely used for a variety of industrial, environmental, and biomedical applications such as electronics, next generation semiconductor, batteries, radient heat materials, and electrochemical biosensors.^178^, ^179^ This ever‐increasing demand for carbon‐based nanomaterials gives rise to the concerns of their potential toxicity to the environment and human health. Especially when suspended in a gaseous phase or liquids, these nanomaterials can lead to an enhanced exposure to workers and consumers, increasing the potential toxicity by entering body through skin pores or eyes.

Back to 1999, Huczko et al. conducted an eye irritation test to study the toxic effects of fullerene matter on Draize rabbit. Among the two eyes of each rabbit, one eye was instilled with 0.2 mL fullerene water suspension while the other eye was set as control. The author found no eye effects within 24, 48, and 72 h, respectively.^180^ Later, in order to evaluate the safety of highly purified fullerenes used in cosmetic industry on eyes, Aoshima et al. performed a toxicity study using laboratory animals, where they found that the highly purified fullerenes could cause conjunctival redness and corneal epithelial defects on rabbits, but these symptoms disappeared in two days after eye‐irritation test.^181^ However, although these researches claimed relatively safe use of fullerene matter, the same results may not be able to directly apply to fullerene nanoparticles, as size generally plays an important role in toxicity. More recently, Ema et al. reported one of their studies on acute ocular irritation of fullerene nanoparticles.^182^ The results showed that no corneal opacity, abnormality of the iris, or chemosis eye were found on experimental rabbits at all the time point after fullerene nanoparticle application. Although conjunctival redness and blood vessel hyperemia were caused by fullerenes at 1 h, 24 h experiment did not exhibit such outcomes. The previous study indeed offered some important information on acute irritation of fullerene nanoparticles, but the study of long term eye toxicity as well as toxicological effects of other fullerene derivatives are still limited. Further study may be needed to clarify the unsolved toxicity regarding fullerenes.

When it comes to carbon nanotubes, different groups tested their eye toxicity using different in vitro or in vivo model. For instance, as shown in a series studies from Liu's group, both SWCNTs and MWCNTs were toxic to human ocular cells. SWCNTs showed a high toxicity to ARPE‐19 cells with a decrease in cell viability, changes in superoxide dismutase (SOD) levels, membrane integrity, and cell apoptosis.^183^ On the other hand, MWCNTs were found to cause a decrease in cell survival rate, an increase in lactate dehydrogenase (LDH) release, and enhancement of ROS generation and apoptotic cells on human retinal pigment epithelium cells.^63^ However, compared to the severe toxicity on cell models, carbon nanotubes show much less adverse effects in experimental animal models. Huczko and Lange found that soot with a high content of SWCNTs had no eye damage in a modified rabbit eye test.^184^ Kishore et al. examined two different sizes of MWCNT ((MWCNT 1:5–8 µm in length with 3–8 nm inside diameter and outside diameter of 140 ± 30 nm; MWCNT 2:1–10 µm in length with 2–6 nm inside diameter and outside diameter of 10–15 nm) for their eye irritation potential both in vivo and in vitro.^185^ As the results showed, only reversible conjunctival redness and discharge were found in rabbits and zero irritation score was found in a Hen Egg Chorion Allantoic Membrane (HET‐CAM) test (a test to investigate eye irritancy in vitro). As no significant toxicity differences were found between two sizes of MWCNTs and the in vivo and in vitro results were in a good correlation, their study further indicated low toxicity of carbon nanotubes on eyes. More recently, Ema et al. investigated eye irritation of two products of SWCNTs and two products of MWCNTs in rabbits, where they found that only one of the MWCNTs (among four) showed a very weak, reversible acute irritant (conjunctival redness and vessel hyperemia) to the eyes 1 h after irritation application.^186^ The other three carbon nanotubes exhibited nonvisible toxic under test conditions.

**Figure 4:**
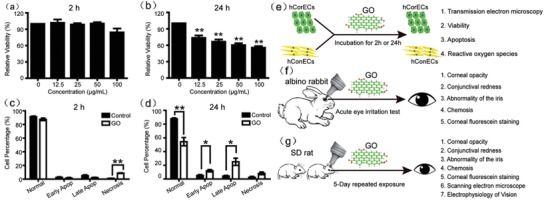
a–d) The toxicity of GO to hCorECs. a,b) WST‐8 assay analysis of viability in hCorECs exposed to GO for 2 and 24 h, respectively. c,d) Flow cytometry analysis of apoptosis in hCorECs exposed to GO (50 mg mL^−1^) for 2 and 24 h, respectively. All the assays were conducted in three independent experiments. Data are presented as mean ± SEM. *p < 0.05. **p < 0.01. e–g) Schematic illustration of the experiments on evaluation of ocular irritation potential of GO exposure using e) in vitro and f,g) in vivo models. Reproduced with permission.^103^ Copyright 2016, Taylor & Francis.

Graphene with its unique functions is widely used in eye‐related biomedical applications nowadays. For instance, due to its outstanding electrical and mechanical properties as well as excellent biocompatibility, graphene is used to coat contact lenses for electromagnetic interference shielding and dehydration protection.^187^ Such direct eye‐contact requires higher safety of graphene application. Previously, Tan et al. used human corneal stromal fibroblasts to study graphene toxicity, they found that graphene showed excellent short‐term biocompatibility with corneal cells and tissues.^188^ However, in practical industrial application, not only graphene but its derivatives such as GO or rGO are also widely used. Therefore, many eye‐toxicity studies also put their focus on graphene derivatives. Yan et al. investigated the toxicity of GO both on human retinal pigment epithelium cells and rabbits.^189^ Their results indicated that GO did not induce any significant toxicity to cell growth and proliferation or damage to rabbit eyes. However, our previous study showed that GO nanoparticles were toxic to primary human corneal epithelium cells and human conjunctiva epithelium cells in a time‐ and dose‐dependent way (**Figure** 4a–d).^103^ In addition, although GO did not cause acute eye irritation on rabbit experiment, short‐term repeated GO exposure to Sprague–Dawley rats induced reversible damage to the eyes via oxidative stress. Our research used both in vitro and in vivo models to study the GO toxicity onto eyes (Figure 4e–g). In consistence to this result, more recently, An et al. studied the ocular toxicity of rGO and GO using both in vitro and in vivo methods.^190^ In their research, rGO exposure did not cause significant ocular toxicity in mice. On the contrary, short‐term repeated GO exposure could result in obvious intraocular inflammation, an incrassated corneal stromal layer, cell apoptosis in cornea, and iris neovascularization in Kunming mice, as well as significant cell death in corneal epidermal cells. Considering the toxic effect of GO, researchers started putting their focus on alleviating the toxicity of GO by using proper functionalization. For instance, we recently investigated the toxicity of PEGylated GO in ocular tissue, and found that the cytotoxicity of PEG‐GO is dependent on oxidation level instead of surface charge.^99^ The exposure of human ocular cells to higher oxidized PEG‐GO could lead to oxidative stress‐related cytotoxicity. Notably, instead of simply testing the “rough” cytotoxicity of PEG‐GO, we also explored the molecular mechanism of PEG‐GO toxicity with different surface charge and oxidation degree using whole‐cell gne expression profiling, it showed that highly oxidized PEG‐GO sample induced ROS‐dependent cytotoxicity through NDUFB9‐mediated pathway. This method may become a powerful tool for studying the toxicity effect of nanomaterials because it provides sufficient information of complex interactions between nanoparticles and biological systems, which could help to better design useful surface modifications to reduce toxicity of graphene‐based nanomaterials for biomedical application.

## 5 Conclusion

In recent years, a dramatic increase in utilization of nanoparticles in food, industry, or cosmetic field has led to an increased concern of their adverse effects on eyes. In this review, we first summarized a few typical ocular disorders upon particulate materials exposure by categories: ocular surface, lens, retina, and optic nerve. As foreign body could induce systematic inflammatory response inside biological bodies, environmental nanoparticles may exert toxicity to all parts of eyes. In addition, we also give a brief introduction of the recent advances of environmental nanoparticle toxicity on eyes. A few number of commonly used nanomaterials in industrial or environmental places (recommended by OECD) are chose to delineate their eye toxicity studies in detail (**Table** 1).

**Table 1:** The list of toxicity studies about commonly used nanomaterials in industrials or environmental places. NP: nanoparticle; SWCNT: single‐walled carbon nanotubes; MWCNT: multiwalled carbon nanotubes; GO: graphene oxide; ROS: reactive oxygen species; SOD: superoxide dismutase; LDH: lactate dehydrogenase

Compound	Biological model	Mechanism	Outcome	Reference
Au NP	Zebrafish eye		Disrupt eye development and pigmentation	127
Ag/Au NP	Cell and tissue culture of mouse retina	Oxidative stress	Apoptosis, Neurotoxic effect, and even visual impairment	62
Ag NP	Bovine retinal endothelial cells	Oxidative stress	Cytotoxicity and apoptosis	128
Ag NP	New Zealand white rabbits		Conjunctival redness, edema, and discharge	129
Ag NP	Guinea pigs		Grade 1 conjunctivae irritation	130
Fe NP	Human corneal epithelial cells	Elevated inflammatory response, cell death‐related pathway indicators and generated mistranscripted RNA	Cell death	139
CeO_2_ NP	Rat retina primary cells, tubby mutant mice, and very low density lipoprotein receptor knockout mouse	Antioxidative effect		149, 154, 155, 156, 157
SiO_2_ NP	Human corneal epithelial cells and Sprague–Dawley rats	Cell membrane damage, cell death, and mitochondrial dysfunction	Corneal injury	50
TiO_2_ NP	Rabbits		Reversible ocular conjunctival redness	168
TiO_2_ NP	New Zealand white rabbits		Ocular surface damage	169
ZnO NP	Rat retinal ganglion cells	Overproducing ROS, caspase 12, decreasing plasma membrane calcium ATPase and bcl 2/caspase 9, disrupting intracellular calcium homeostasis	Cell death	170, 171, 172
ZnO NP	Sprague–Dawley rats		Retinopathy	67
Fullerene	Rabbit		Conjunctival redness and corneal epithelial defects	181
Fullerene	Rabbits		Conjunctiva redness and blood vessel hyperemia	182
SWCNT	ARPE‐19	Changes in SOD levels, membrane integrity and cell apoptosis	Cell death	183
MWCNT	Human retinal pigment epithelium cells	increase in LDH release, ROS generation and apoptosis	Decrease in cell survival rate	63
MWCNT	Rabbit		Conjunctival redness/discharge and vessel hyperemia	185, 186
GO NP	Primary human corneal epithelium cells and human conjunctiva epithelium cells; Sprague–Dawley rats	Oxidative stress	Cell death	103
GO NP	Kunming mice and corneal epidermal cells	Inflammation and apoptosis	Incrassated corneal stromal layer and iris neovascularization	190

Efforts may have been put into the eye toxicity examination of some nanoparticles, it is far from enough. There are still limitations regarding current studies.

1. In nanoparticle eye toxicity studies, different research groups utilized different biological models including cell lines, aquatic organisms (e.g., embryonic zebrafish), and whole animals (e.g., rabbits or rodents). Sometimes, even using the same biological model, taking cell lines for example, the type of cell lines, culturing conditions, and incubation time also vary from group to group.^50^, ^162^ Considering the nature of nanoparticles during toxicity examinations, however, it is irrational to compare results from different research groups and determine whether the obtained toxicity results are physiologically relevant. It is also difficult for us to get a systematic toxicity conclusion of certain nanoparticles from various different research results. Therefore, the standard strategies and interpretation of research outcomes are called for the correct understanding of nanomaterial toxic effects to eyes.^191^
2. Although some researches claim that nanoparticle could cause reversible toxicity to eyes, almost all of them fail to testify such conclusion in a long‐term repeated study. Most workers or residents in nanomaterial‐workplace expose their eyes to nanomaterials for years. Therefore, only weeks or months repeated study is not potent to claim the safety of nanomaterials to eyes. Consequently, long‐term toxicity studies are still needed to claim the safe use of nanomaterials.
3. As seen from the current outcome we have obtained, the toxicity effect of nanoparticle on eyes might be worse than we imagine. Starting with mild discomfort on eyes, conditions could progress to pretty severe nerve or visual impairment, or even loss of vision. Besides, it is also proposed that, in addition to causing direct damage to eyes, the exposure of nanoparticles to eye could also induce some inner damage. For instance, silver and TiO_2_ nanoparticles could translocate into central nervous system though eye‐to‐brain pathways, which could induce neuroinflammation as well as other problems, such as entering blood.^25^, ^192^ Such blood‐assisted distribution can lead to a severe problem. As nanomaterials generally cannot be metabolized and only ultrasmall nanoparticles have a potential to be excreted by renal pathway, most nanomaterials tend to accumulate in various tissues including spleen, liver, or lymph nodes.^193^ From this point of view, nanoparticle exposure could not only induce adverse effect to eyes, but also expose potential safe concern on other parts of body through ocular absorption. Further studies regarding their inner toxicity examination should also be carried out to realize a comprehensive safety study of eye‐absorbed nanomaterials.

Taken together, eye safety in nanomaterials industry is of equal importance as skin or pulmonary safety. The study of their toxicity can not only help us understand the haphazardness of nanoparticle use, but also give us the hint on how to better protect ourselves in such environment. It is reported that adequate eye protection could prevent 90% of work‐related eye injuries.^194^ In order to protect eyes from particulate dust and vapors, goggles with side shields, and proper cleaning solutions for contact lens users should be provided in the nanomaterials workplaces. With better understanding of the nanoparticle toxicity to eyes, we can make a safer environment for nanomaterial contacts.

## Conflict of Interest

The authors declare no conflict of interest.
